# Intermittent Fasting Dietary Restriction Regimen Negatively Influences Reproduction in Young Rats: A Study of Hypothalamo-Hypophysial-Gonadal Axis

**DOI:** 10.1371/journal.pone.0052416

**Published:** 2013-01-29

**Authors:** Sushil Kumar, Gurcharan Kaur

**Affiliations:** Department of Biotechnology, Guru Nanak Dev University, Amritsar, Punjab, India; National Institutes of Health, United States of America

## Abstract

Nutritional infertility is very common in societies where women fail to eat enough to match their energy expenditure and such females often present as clinical cases of anorexia nervosa. The cellular and molecular mechanisms that link energy balance and central regulation of reproduction are still not well understood. Peripheral hormones such as estradiol, testosterone and leptin, as well as neuropeptides like kisspeptin and neuropeptides Y (NPY) play a potential role in regulation of reproduction and energy balance with their primary target converging on the hypothalamic median eminence-arcuate region. The present study was aimed to explore the effects of negative energy state resulting from intermittent fasting dietary restriction (IF-DR) regimen on complete hypothalamo-hypophysial-gonadal axis in Wistar strain young female and male rats. Significant changes in body weight, blood glucose, estrous cyclicity and serum estradiol, testosterone and LH level indicated the negative role of IF-DR regimen on reproduction in these young animals. Further, it was elucidated whether serum level of metabolic hormone, leptin plays a mechanistic role in suppressing hypothalamo-hypophysial-gonadal (HPG) axis via energy regulators, kisspeptin and NPY in rats on IF-DR regimen. We also studied the effect of IF-DR regimen on structural remodeling of GnRH axon terminals in median eminence region of hypothalamus along with the glial cell marker, GFAP and neuronal plasticity marker, PSA-NCAM using immunostaining, Western blotting and RT-PCR. Together these data suggest that IF-DR regimen negatively influences reproduction in young animals due to its adverse effects on complete hypothalamus-hypophysial-gonadal axis and may explain underlying mechanism(s) to understand the clinical basis of nutritional infertility.

## Introduction

Animals adjust their energy requirements when food is scarce or have a very high acquisition cost [1] and under metabolic stress, all the animals invest energy in survival rather than reproduction. Although the reproductive system is sensitive to change in energy status [1] but the physiological mechanisms explaining the link between energy balance and reproduction are not clearly understood. When food supply is insufficient to meet metabolic demands, puberty is delayed, and also ovulation and estrous cycle are suppressed [2], [3]. Food restriction has been known to exert negative influence on luteinizing hormone (LH) pulsatility in prepubertal or cycling gilts [4], on ovarian development [5] and decrease in gonadotropin concentration in humans [6]. Both gonadotropin releasing hormone (GnRH) pulse generator system and gonadotropin secretion are inhibited by food restriction and the gonadotropin effect for folliculogenesis may not be sufficient [7], [8]. Understanding the physiological basis of joint regulation of energy balance and reproductive function could help to design better strategies for maintaining reproductive health.

Circulating leptin plays a potential role in nutritional signaling to the brain and in the regulation of reproduction and energy balance with its primary target pathway in the hypothalamic arcuate nucleus [9]. Leptin regulates a neuronal population mainly located at the arcuate, expressing either orexigenic (NPY and agouti related peptide, AgRP) and anorexigenic (proopiomelacortin, POMC and cocaine and amphetamine-regulated transcript, CART) peptides [10]. Leptin alters the hypogonadotrophic state in lean animals by increasing POMC gene expression, and transport and acylation of melanocortins in the brain [11]. Food restriction has been shown to modulate NPY gene expression during the phase of low leptin levels [12] and action of leptin on LH secretion is also dependent upon the availability of glucose [13]. Recent study shows that increase in hypothalamic NPY gene expression by caloric restriction might result from the coordinate action of several factors including the decrease in serum leptin and insulin concentration [14]. Few other reports showed that increase in NPY level may stimulate feeding behaviour and inhibit GnRH release during some periods of feeding cycle [15], [3].

Kisspeptin and NPY neurons are considered good candidates in linking pertuberations in energy balance with alterations in the activity of reproductive axis. NPY, a potent orexigenic neuropeptide involved in the neuroendocrine control of reproductive axis, may metabolically regulate KISS-1 expression in the hypothalamus [16], [17]. Several reports suggest the role of KISS-1/kisspeptin/GPR54 system as a major gatekeeper of GnRH neurons and involved in metabolic control of reproductive axis [18], [19]. Short term fasting (72 h) in pubertal animals evoked a decrease in the expression of KISS-1 mRNA which was associated with reduction in circulating leutinizing hormone level in both the male and female rats [18].

Fluctuating physiological conditions reversibly alter the structural relationship among the neurons and glial cells in the hypothalamus and the functional pathways over which the information is transmitted to the neuroendocrine neurons [20], [21]. Homopolymer of ∞-2,8 linked sialic acid i.e. PSA residues expressed on the extracellular domain of neural cell adhesion molecule (PSA-NCAM) is known to have anti-adhesive role and intervenes in dynamic neuron-glia interactions [20], [22], [23]. Previous studies have revealed direct GnRH neuro-haemal contact on the day of proestrous [20], [24], whereas, astro-glial cells extend their processes towards the pericapillary space and block contact of GnRH terminals with perivascular space on diestrous day. Recently we have reported that removal of PSA from NCAM *in vivo* also blocked the retraction of glial processes and decreased GnRH neuron remodeling to secrete GnRH in perivascular space [25]. The co-localization of PSA-NCAM with GnRH in the external zone of median eminence (ME) in proestrous phase control rats suggest its permissive role in the regulation of GnRH release in perivascular space by allowing retraction of astro-glial processes.

The current study was aimed to understand the cellular and molecular mechanisms that may link energy status with reproduction in animals on IF-DR regimen for 12 weeks. 3–4 months old young adult rats of both the sexes were used for this study. After assessing the effect of IF-DR regimen on estrous cycle and serum level of estradiol and LH in female and testosterone and LH level in male animals, the study was further extended to explore the serum level of leptin hormone and hypothalamic expression of NPY and kisspeptin neuropeptides which are known to mediate nutritional signaling to regulate reproduction and energy balance at hypothalamic level. Previous studies from our lab and others have reported that GnRH axon terminals and associated glial cells in female rats undergo morphological remodeling to facilitate GnRH release near the perivascular space in median eminence during the proestrous phase of the reproductive cycle [24], [26], [27]. To further investigate whether change in energy status due to IF-DR regimen negatively influence the reproductive function in rats at the level of these central regulatory mechanisms as well, we also explored the effect of IF-DR regimen on GnRH axon terminals remodeling in ME region of hypothalamus in both male and female rats alongwith the expression of plasticity markers such as PSA-NCAM and GFAP. To ascertain whether these changes are also regulated at post translational level, polysialyltransferase (PST), GnRH and GFAP mRNA expression was studied in IF-DR and ad libitum (AL) fed male and female rats. Although, several previous studies have focused on the influence of caloric as well as food restriction on gonadotropin secretion but to the best of our knowledge, this is the first comprehensive report to elucidate the negative impact of IF-DR regimen on reproduction at all levels of HPG axis in young adult rats of both the sexes. The study may help to understand the cellular and molecular basis of clinically recognized menstrual irregularities observed in women due to inadequate food intake.

## Materials and Methods

### Ethics Statement

All animal work was approved by the Animal Ethics Committee of the Guru Nanak Dev University, Amritsar, Punjab, India.

### Experimental Animals

Wistar strain young adult male and virgin cycling female albino rats in the age group of 3–4 months and weighing 150–200 gm were used for these experiments. Animals were procured from Disease free small animal house, CCSHAU, Hisar and were housed 3 per cage under 12 h light and 12 h dark conditions. Animal care and procedures were followed in accordance with the guidelines of Animal Ethical Committee, Guru Nanak Dev University, Amritsar (permit number for this study: 2270/ZD). The estrous cycle was monitored by daily inspection of vaginal cytology in female rats and the paradigm of IF-DR involved periodic fasting, in which 3 months old wistar strain male and female albino rats were deprived of food for a full day, every other day, and fed ad libitum (AL) on the intervening day for 12 weeks. Food was provided and removed at 10.00 am every day. Previous studies have shown that rats and mice maintained on such an every-other-day feeding regimen consume about 30–40% less calories over time and live longer than AL fed animals [28]. The rats of age matched control group were fed ad libitum and used as control. A group of IF-DR female animals was put on refeeding (DR-R) for 8 weeks. AL fed, IF-DR and DR-R rats were given water ad libitum. The body weight and blood glucose level were recorded every fifteenth day in IF-DR and age matched AL fed rats.

### Ovarian Histology

After 12 weeks of IF-DR regimen, one group of animals (n = 6) alongwith control female rats (n = 6) and DR-R in pro-estrous phase were sacrificed via anesthetic overdose with sodium pentobarbital (100 mg/Kg). The animals were weighed before sacrificing, brain tissue was removed for immunohistochemistry and the blood sample was used for serum isolation. Ovaries were removed from IF-DR, AL fed and DR-R animals and weighed before putting them into formalin solution for fixation. For microscopial evaluation of ovaries, the mid-ovarian sections were cut for studying follicular morphology by Eosin and Haematoxylin staining.

### ELISA

#### Sample collection

Blood sample was collected at the end of the fasting day after completion of 12 weeks of IF-DR regimen and blood sample from age matched AL fed animals (male and female) was collected after the overnight fasting of these animals and allowed to clot for 30 min. The samples were then centrifuged for 15 min at 5000 rpm. Serum was separated and immediately assayed for leptin, LH, Estradiol and Testosterone ELISA.

#### Leptin and LH ELISA

The serum leptin and LH concentration from both female and male rat (n = 6 each in duplicate) groups was determined using rat Leptin ELISA kit (Millipore, USA) and rat LH ELISA kit (Cusabio, China) according to the manufacturer’s instructions using Multiscan Ascent Microplate Reader (Thermo Electron Corporation). The concentration of serum leptin (ng/ml) and LH (mlU/ml) was then calculated using kit provided standards and Sigma Stat software.

#### Estradiol and testosterone assay

Estradiol and testosterone in female and male rats, respectively was assayed in IF-DR and control groups (n = 6 each in duplicate) using ELISA kits from Cayman Chemical Company, Ann Arbor, MI according to the manufacturer’s instructions. All the readings were calculated for the preparation of data prior to the graphical analysis. Standard curve was plotted using kit provided standards and, estradiol and testosterone level are presented in pg/ml.

### Immunostaining of NPY and Kisspeptin

Immunostaining of NPY and kisspeptin in (median eminence-arcuate) ME- ARC region of hypothalamus from AL fed and IF-DR young male and female rats (n = 6 each) was performed by DAB method described earlier [29]. Rats were deeply anaesthetized (120 mg/kg pentobarbital) and perfused transcardially with 100 ml of normal saline, followed by 200 ml of 4% paraformaldehyde in 0.01M phosphate buffer, pH 7.4. Brains were post fixed for 24 h in 4% paraformaldehyde and cryopreserved in 20% and 30% sucrose in phosphate buffer each for 24 h at 4°C. 30 µm thick coronal sections were cut using cryostat microtome (Thermo) and permeabilized with 0.3% Triton X-100 in Phosphate buffer saline (PBST). Sections were incubated with anti-NPY (1∶500, Chemicon) and anti-kisspeptin10 (1∶500, Chemicon) primary antibodies in 0.1% Triton X-100 and 5% NGS for 48 hours at 4°C in humid chamber. Biotinylated secondary antibody anti-rabbit IgG for NPY and kisspeptin (1∶400, sigma) in 0.1% PBST was applied for 2 hours at room temperature. Sections were incubated with extravidin-peroxidase (1∶400, sigma) in 0.01% PBST for 2 hours and then with diaminobenzidine (DAB, Sigma) for 15–20 min. Sections were air dried overnight followed by treatment with 50%, 70%, 90% and 100% ethanol. Tissue sections were then cover slipped using the DPX permanently. Images were captured under (10X) using brightfield option in Nikon Eclipse (E600) fluorescent microscope and were analyzed using image pro-plus software version 4.5.1 from the media cybernetics.

### Immunofluorescent Staining of GnRH, PSA-NCAM and GFAP

Immunofluorescence labelling of GnRH, PSA-NCAM and GFAP in ME-ARC region of hypothalamus from AL fed and IF-DR young male and female rats (n = 6 each) was done according to the protocol described earlier [25]. 30 µm thick coronal sections and incubated with primary antibodies anti-PSA-NCAM (1∶500, AbCys, France), anti-GFAP (1∶500, sigma) and anti-GnRH (1∶2000, sigma). Secondary antibody (Alexa Fluor 488 and 568, Invitrogen) was applied for 2 h at room temperature and then mounted with anti-fading reagent (Fluoromount, Sigma) and observed under the microscope (Nikon A1RConfocal).

### Data Analysis

Images were acquired using Nikon A1R Confocal Microscope and the images were analyzed using Image Pro-plus TM software version 4.5.1 from the Media cybernetics. The intensity of immunoreactivity was quantified in randomly selected fields in each section using the count/size command. Five consecutive sections each from 5 to 6 animals in all groups were used for data analysis. An area of interest (AOI) was selected and placed within region on each section and density measurement was made for staining intensity. All the slides were coded for their group assignment and code was not broken till the intensity was measured. An examiner blind to the group assignment of each animal did density measurement. Results are expressed as the average of staining intensity and presented as the mean ±S.E.M. Differences between means were determined using student t-test, and p<0.05 was considered statistical significant.

The co-localization of GnRH and PSA-NCAM was done using the co-localization command of the Image Pro-Plus software. This command measures the co-localization coefficient of two or more molecules (tagged with different fluorescent dyes) in precisely the same space.

### Western Blotting of PSA-NCAM and GFAP

Western blotting was done according to the protocol described earlier [30]. Tissue sample for Western blot study was collected from IF-DR animals which were sacrificed by cervical dislocation. Briefly, ME-ARC region of hypothalamus (n = 5 each for control and test) was microdissected and pooled from AL fed and IF-DR young male and female rats. Protein estimation was done by Bradford’s method and supernatant was mixed with 6X sample buffer. Protein lysate (25–30 µg) was resolved on 8–10% SDS-PAGE followed by transfer onto a PVDF membrane (Hybond-P) using the semidry Novablot system (Amersham Pharmacia). Subsequently, membranes were probed with anti-PSA-NCAM (1∶2000) and anti-GFAP (1∶5000) monoclonal antibodies. Immunoreactive bands were visualized using ECL Plus Western blot detection system (Amersham Biosciences). In order to account for potential variations in protein estimation and sample loading, expression of each protein was compared to that of α-tubulin.

### Kisspeptin, NPY, GnRH, PST and GFAP mRNA Expression by RT-PCR

Tissue from ME-ARC region of hypothalamus (n = 5) was pooled from AL fed and IF-DR young male and female rats to isolate RNA using TRI reagent (Sigma) according to manufacturer’s instructions. The expression of kisspeptin, NPY, GnRH, PST and GFAP mRNA was quantified by semi-quantitative reverse transcriptase–polymerase chain reaction (RT**–**PCR) analysis according to the protocol as described earlier [31]. Equal amounts of RNA were used for cDNA synthesis in 20 µl reactions containing 200U M-MLV reverse transcriptase, 4 µl 5X first strand buffer (Fermentas), 5 µg of total RNA, 1 mM each of dNTPs (Fermentas), 20 units of ribonuclease inhibitor (Sigma), and 250 ng pd(N)_6_ random hexamers (Fermentas). 2 µl of cDNA was amplified in a 50 µl PCR reaction mixture containing two units Taq polymerase, 5 µl 10X PCR buffer, 3.0 µl of 25 mM MgCl_2_ (Sigma), 1 µl of 10 mM dNTP mix (Fermentas), and 20 picomoles of respective primers as listed in Table 1. Cycling conditions comprised of an initial denaturation of 3 min at 94°C followed by 35 cycles of amplification (at 94°C for 40 sec, 55°C for 45 sec and 72°C for 1 min) and final elongation step at 72°C for 10 min. To control the PCR reaction components and the integrity of the RNA, 2 µl of each cDNA sample was amplified separately for β-actin specific primers.

**Table 1:** Primer sequences used for semi-quantitative RT-PCR.

Sr. No.	mRNA	Primer Sequence	Expected Product Size
1.	Kisspeptin	F5′AGCTGCTGCTTCTCCTCTGT3′	320 bp
		R5′AGGCTTGCTCTCTGCATACC3′	
2.	NPY	F5′GGGGCTGTGTGGACTGACCCT 3′	150 bp
		R5′GATGTAGTGTCGCAGAGCGGAG 3′	
3.	GnRH	F5′GGCAAGGAGGAGGATCAA A3′	124 bp
		R5′CCAGTGCATTACATCTTCTTCTG3′	
4.	PST	F5′TAAGGTGCAATCTAGCTCCTGTGGTGG3′	474 bp
		R5′GCATCCTGTGAGGACTGGCGTTGGAAA3′	
5.	GFAP	F5′GGCGCTCAATGCTGGCTTCA3′	326 bp
		R5′TCTGCCTCCAGCCTCAGGTT3′	
6.	β – Actin	F5′TCACCCACACTGTGCCCATCTACG A3′	285 bp
		R5′CAGCGGAACCGCTCATTGCCAATGG3′	

## Results

### Body Weight, Blood Glucose Level, Estrous Cycle and Ovarian Histology in AL Fed and IF-DR Rats

**Figure 1:**
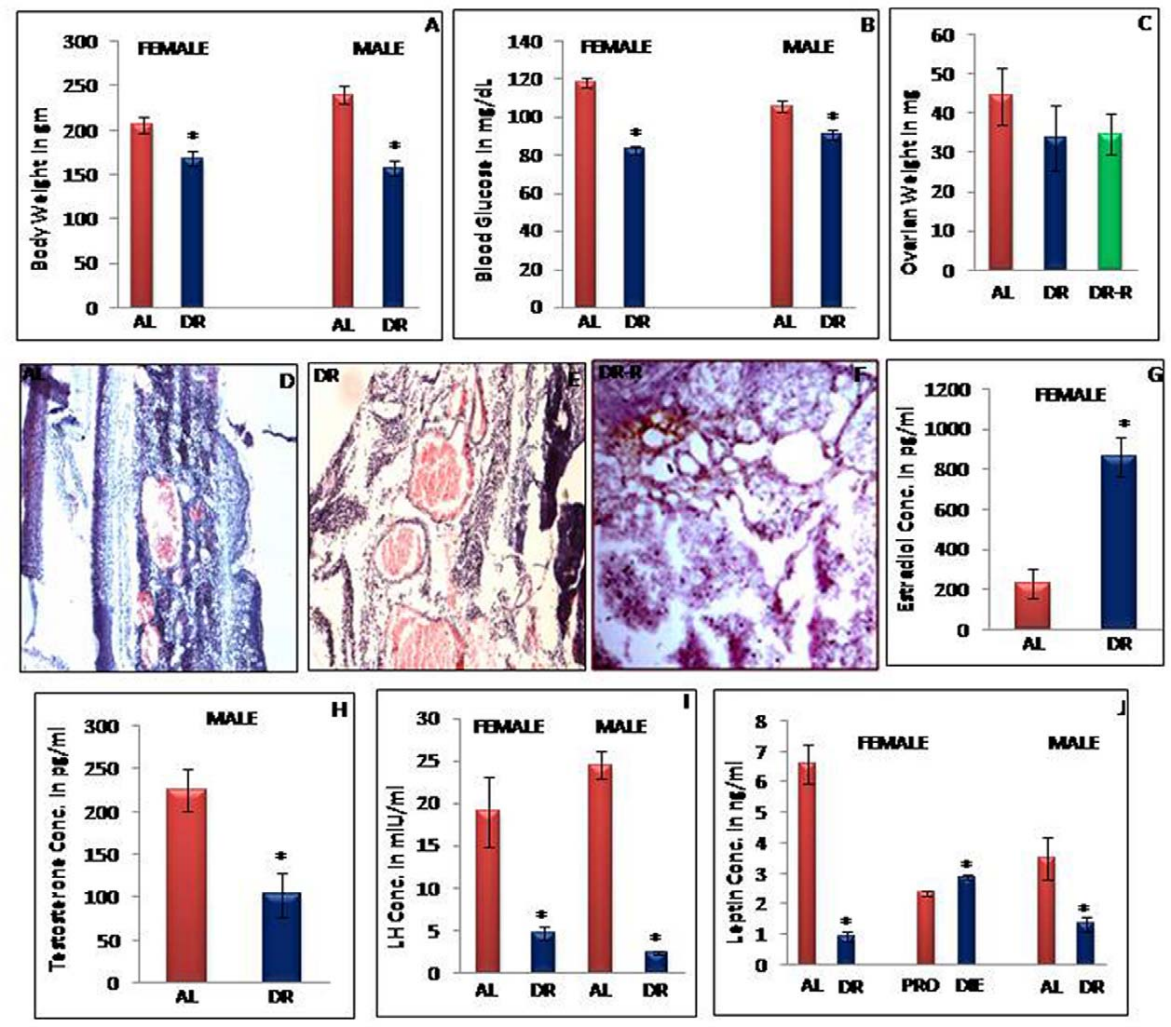
Effect of IF-DR regimen on body weight, ovarian histology, blood glucose, serum leptin, LH, estradiol and testosterone level. Comparison of Body weight gain (in gm), blood glucose (in mg/dL), ovarian histology, ovarian weight (in mg), Serum estradiol and testosterone (in pg/ml), LH (in mlU/ml), leptin (in ng/ml) level in ad libitum fed (AL), Intermittent fasting dietary restricted (IF-DR), DR-R female and male rats (n = 6 each). All the parameters were observed after 12 weeks of alternate day feeding of 3–4 months old and (B) Blood glucose level were significantly lower in IF-DR animals of both sexes as compared to AL group. (C) Reduced ovarian weight in IF-DR female rats. (D–F) Large sized corpora lutea and fibrous tissue were seen in ovarian sections of IF-DR as compared to AL fed and DR-R female rats. (G) Higher level of serum estradiol in IF-DR as compared to AL female rats. (H) Reduced testosterone concentration in IF-DR male rats as compared to control animals. (I) Serum LH level was reduced in IF-DR animals of both the sexes. (J) Serum leptin level was lower in IF-DR animals of both sexes as compared to their respective control group. Diestrous female rats showed higher level of serum leptin than pro-estrous phase rats. Values are mean ±SEM. *pValue<0.05.

After 10–15 days of IF-DR, female rats showed disrupted estrous cycle and animals remained in the diestrous phase for next 10–11 weeks, whereas, control rats and DR-R rats showed regular estrous cycle of 4–5 days and were sacrificed in the afternoon of proestrous phase. Fig. 1A represents change in body weight after 12 weeks of IF-DR regimen. There was significant decrease in body weight of dietary restricted female and male rats as compared to their respective AL fed controls. Blood glucose level was also decreased significantly in IF-DR female and male rats as compared to control group (Fig. 1B). After 12 weeks of dietary restriction, ovarian weight was observed to be reduced in IF-DR and DR-R rats as compared to control group (Fig. 1C), although the change was not statistically significant. Ovarian sections showed large sized corpora lutea and the fibrous tissue in the central cavity in IF-DR female rats (Fig. 1E). The ovarian sections from AL fed control (Fig. 1D) and DR-R (Fig. 1F) rats showed degenerated corpora lutea with central vacuole which is the marker of proestrous phase.

### LH, Estradiol and Testosterone Level in IF-DR and Control Rats

Serum LH, estradiol (female rats) and testosterone (male rats) levels were assayed from IF-DR and control rats. Serum LH level was significantly reduced after 12 weeks of IF-DR regimen in both female and male rats as compared to AL fed controls (Fig. 1I). Further, serum estradiol concentration in female rats on IF-DR regimen was found to be significantly higher (Fig. 1G) whereas, serum testosterone level in male IF-DR rats was reduced (Fig. 1H) as compared to their respective AL fed control rats.

### IF-DR Regimen Affects Serum Leptin Level

Serum was collected from IF-DR, AL fed, proestrous and diestrous animals and leptin level was measured by ELISA. IF-DR regimen resulted in altered pattern of serum leptin level in both male and female rats. The results indicate that IF-DR significantly decrease serum leptin level in female rats as compared to control group. Moreover, IF-DR regimen also caused mild reduction in serum leptin level in IF-DR as compared to AL fed male rats (Fig. 1J). Furthermore, serum leptin concentration was increased in diestrous as compared to proestrous female rats (Fig. 1J).

### IF-DR Regimen Enhanced the Expression of NPY in ME-ARC Region of Hypothalamus

**Figure 2:**
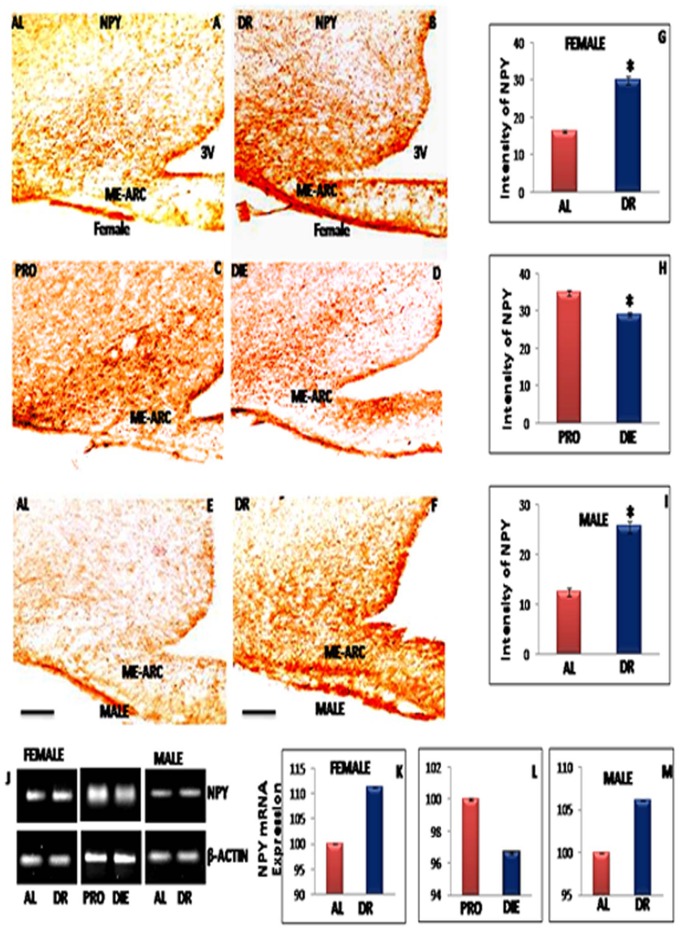
Representative immunohistochemical staining and RT-PCR data of NPY. Immunostained images of 30 µ thick coronal sections from median eminence-arcuate (ME-ARC) region of female rats in pro-estrous (PRO) and diestrous (DIE) phase, ad libitum fed (AL) and Intermittent fasting dietary restricted female and male (n = 5 each) rat brain. The DAB staining in the ME-ARC region for NPY is shown for AL and IF-DR female rats (A and B), PRO and DIE phase female rats (C and D), and AL and IF-DR male rats (E and F), respectively. NPY-ir was higher in IF-DR rats as compared to their corresponding control (AL) female (B and A) and male (F and E) rats. NPY-ir was reduced in DIE phase as compared to PRO phase in female rats. (J) Median eminence-arcuate region NPY mRNA expression was enhanced in IF-DR male and female rats as compared to control group; however NPY mRNA expression was reduced in diestrous phase as compared to pro-estrous phase female rats. (G, H, I, K, L and M) depicts relative intensity measurement ±SEM of NPY-ir, and mRNA expression performed by an observer blind to the experiments. *pValue<0.05. Scale bar = 100 µ (A–F).

Rats on IF-DR regimen were sacrificed on the day of fasting for both male and female rats, whereas, AL fed rats were sacrificed on pro-estrous day afternoon. Further hypothalamic NPY expression was studied in dietary restricted diestrous and AL fed proestrous alongwith virgin female rats in diestrous and proestrous phase of estrous cycle. Immunohistochemical study of NPY in arcuate region of hypothalamus indicated that IF-DR regimen resulted in up regulation of NPY expression in hypothalamus which was found to be higher in IF-DR female (Fig. 2B) and male (Fig. 2F) rats as compared to the respective control groups (Fig. 2A, 2E). The opposite pattern was observed in young cycling female rats. NPY-ir was higher in proestrous rats (Fig. 2C) as compared to diestrous (Fig. 2D) animals. The staining intensity analysis also depicted significant increase in NPY-ir in arcuate region of IF-DR animals as compared to control group (Fig. 2G, 2I). The results of immunohistochemical localization of NPY were further confirmed by RT-PCR. Fig. 2J shows increased NPY mRNA expression in IF-DR (Fig. 2K, 2M) and proestrous group as compared to AL fed and diestrous rats (Fig. 2L).

### Effect of IF-DR Regimen on the Expression of Kisspeptin in ME-ARC Region of Hypothalamus

**Figure 3:**
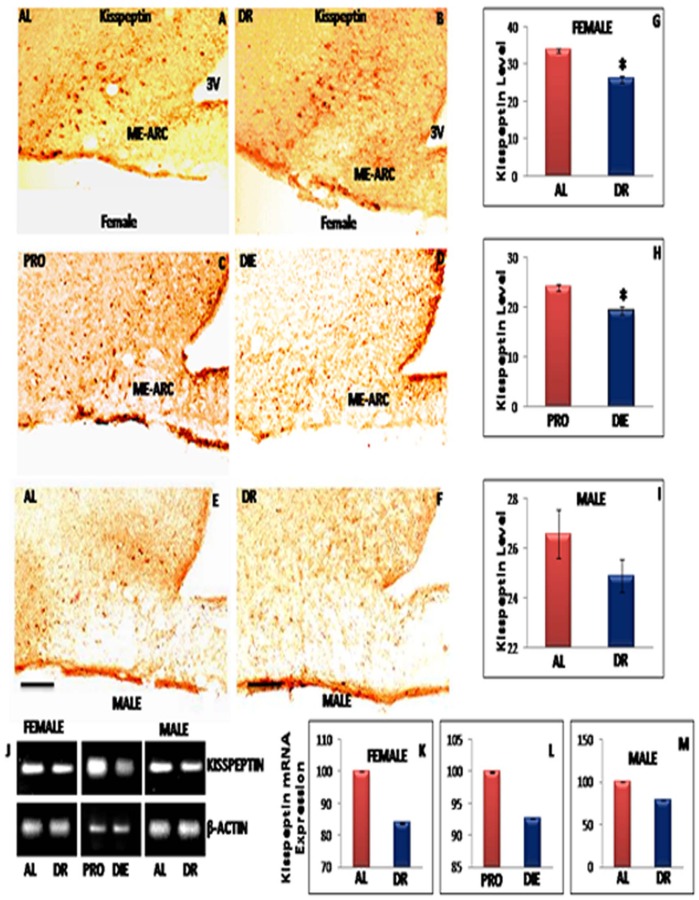
Representative immunohistochemical staining and RT-PCR data of kisspeptin. Immunostained images of 30 µ thick coronal sections from median eminence-arcuate (ME-ARC) region of female rats in pro-estrous (PRO) and diestrous (DIE) phase, ad libitum fed (AL) and Intermittent fasting dietary restricted female and male (n = 5 each) rat brain. The DAB staining in the ME-ARC region for Kisspeptin is shown for AL and IF-DR female rats (A and B), PRO and DIE phase female rats (C and D), and AL and IF-DR male rats (E and F), respectively. Kisspeptin-ir was reduced in IF-DR rats as compared to their corresponding control (AL) female (B and A) and male (F and E) rats. Kisspeptin-ir was also reduced in DIE phase as compared to PRO phase in female rats. (J) Median eminence-arcuate region Kisspeptin mRNA expression was reduced in IF-DR male and female rats as compared to control group; Also, Kisspeptin mRNA expression was reduced in diestrous phase as compared to pro-estrous phase female rats. (G, H, I, K, L and M) depicts relative intensity measurement ±SEM of kisspeptin-ir and mRNA expression level, performed by an observer blind to the experiments. *pValue<0.05. Scale bar = 100 µ (A–F).

Immunohistochemical localization showed decrease in the kiss-ir in female rats maintained on IF-DR regimen (Fig. 3B) than control group (Fig. 3A). Kiss-ir was found to be higher in proestrous (Fig. 3C) as compared to diestrous (Fig. 3D) rats. Mild reduction was observed in IF-DR (Fig. 3F) than AL fed (Fig. 3E) male rats. Quantitative analysis of immunostaining results revealed significant decrease in hypothalamic kisspeptin level during different stages of cyclicity and in IF-DR female rats (Fig. 3G, H). RT-PCR results also indicated decreased kisspeptin mRNA expression in IF-DR (Fig. 3J, K) and diestrous (Fig. 3J, L) female rats, which is also reflected in intensity analysis of kisspeptin mRNA expression in male rats (Fig. 3J, M).

### GnRH Immunoreactivity in ME Region of Hypothalamus

**Figure 4:**
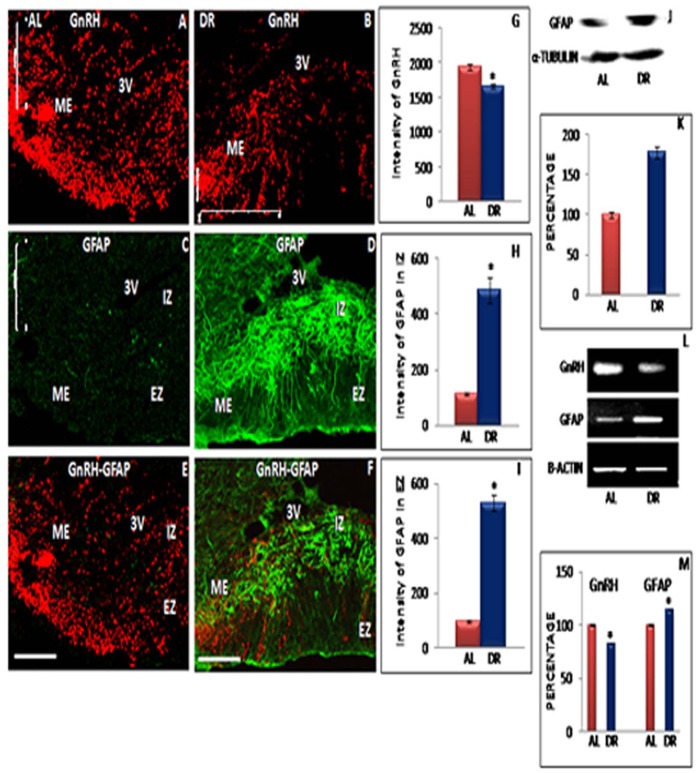
Representative immunofluorescent staining, Western blot and RT-PCR data of GnRH and GFAP in female rats. Confocal immunofluorescent images of 30 µ thick coronal sections from median eminence (ME) region of ad libitum fed (AL) and Intermittent fasting dietary restricted (IF-DR) female (n = 5 each) rat brain. The immunostaining in the ME region for GnRH and GFAP is shown for GnRH (A and B), GFAP (C and D) as well as dual immunofluorescence of GnRH and GFAP (E and F) for AL and IF-DR animals, respectively. GnRH-ir was reduced in IF-DR rats as compared to their corresponding control AL fed rats (B and A). GFAP immunostaining is visible in both internal and external zone of ME in IF-DR animals (D), whereas, GFAP-ir was reduced and restricted to internal zone of median eminence in AL group (C). Further (E) and (F) represent colocalization of GFAP and GnRH. (J) Western blot hybridization for GFAP and α-tubulin from median eminence region of AL and IF-DR female rats. (L) RT-PCR results for GnRH, GFAP and actin from median eminence region of AL and IF-DR female rats. (G, M) depicts relative intensity measurement ±SEM of GnRH immunofluorescence and relative optical density, performed by an observer blind to the experiments. (H and I) depict relative intensity measurement ±SEM of GFAP immunofluorescence from internal and external zone of ME. (K and M) depict relative optical density ±SEM of GFAP western blot analysis and mRNA expression from median eminence region. *pValue<0.05. Scale bar = 60 µ (A–F).

**Figure 5:**
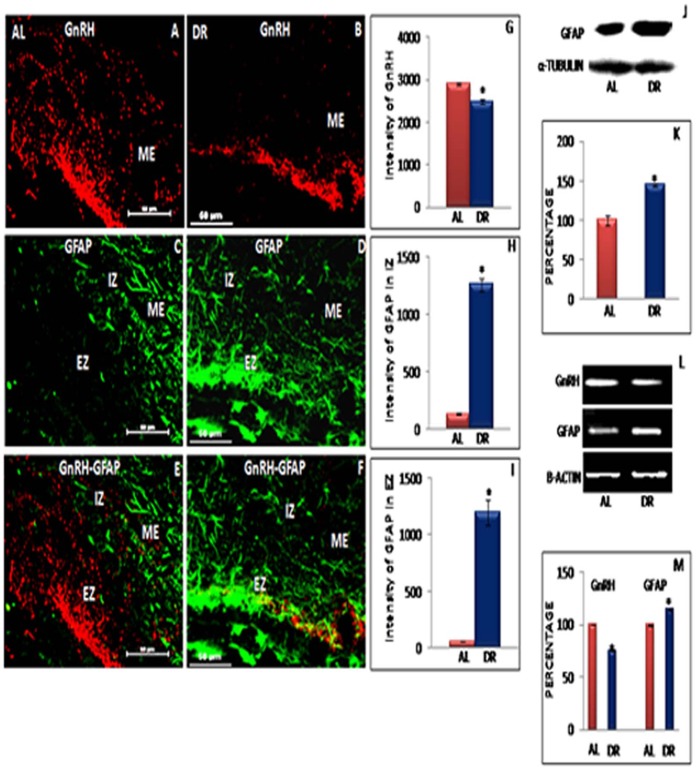
Representative immunofluorescent staining, Western blot and RT-PCR data of GnRH and GFAP in male rats. Confocal immunofluorescent images of 30 µ thick coronal sections from median eminence (ME) region of ad libitum fed (AL) and Intermittent fasting dietary restricted (IF-DR) male (n = 5 each) rat brain. The immunostaining in the ME region for GnRH and GFAP is shown for GnRH (A and B), GFAP (C and D) as well as dual immunofluorescence of GnRH and GFAP (E and F) for AL and IF-DR animals, respectively. GnRH-ir was reduced in IF-DR rats as compared to their corresponding control AL fed rats (B and A). GFAP immunostaining is visible in both internal and external zone of ME in IF-DR animals (D), whereas, GFAP-ir is reduced and restricted to internal zone of median eminence in AL group (C). Further (E) and (F) represent colocalization of GFAP and GnRH. (J) Western blot hybridization for GFAP and α-tubulin from median eminence region of AL and IF-DR male rats. (L) RT-PCR results for GnRH, GFAP and actin from median eminence region of AL and IF-DR male rats. (G, M) depicts relative intensity measurement ±SEM of GnRH immunofluorescence and relative optical density, performed by an observer blind to the experiments. (H and I) depict relative intensity measurement ±SEM of GFAP immunofluorescence from internal and external zone of ME. (K and M) depict relative optical density ±SEM of GFAP western blot analysis and mRNA expression from median eminence region. *pValue<0.05. Scale bar = 60 µ (A–F).

Dietary restricted animals showed decline in GnRH expression as well as marked morphological changes in GnRH axon terminals in the ME region. Fig. 4 and 5 present expression of GnRH-ir in ME region of IF-DR female (Fig. 4B) and male rats (Fig. 5B) as compared to their respective control groups (Fig. 4A and 5A). On quantitative analysis, GnRH immunostaining was seen to be significantly reduced in IF-DR female (Fig. 4G) and male (Fig. 5G) rats as compared to AL fed animals. Moreover, the GnRH immunostaining was higher and the axon terminals were seen to extend thin processes towards the perivascular space in AL fed animals indicating the site of active GnRH secretion. The percentage change observed in GnRH staining intensity was comparable in female and male rats. The GnRH immunostaining was further confirmed by quantitative estimation of GnRH mRNA expression. The RT-PCR results also confirm reduced expression of GnRH in IF-DR female (Fig. 4L) and male (Fig. 5L) rats as compared to AL fed animals. The mean percentage value of GnRH to β-actin was found to be significantly decreased in IF-DR female (Fig. 4M) and male (Fig. 5M) rats.

### Expression of GFAP in IF-DR and Proestrous Control Rats

The astro-glial cells were seen to lose their star shaped structure which may be due to retraction of their processes surrounding GnRH axon terminals. Co-expression of GnRH and GFAP in AL fed and IF-DR rats is shown in Fig. 4 and 5. The present data showed extended processes of astro-glial cells surrounding the GnRH axon terminals in ME region of IF-DR female (Fig. 4D) and male rats (Fig. 5D). GnRH axons were co-distributed with the glial elements in both internal and external zone of ME in the IF-DR female and male rats (Fig. 4F and 5F), whereas, GFAP expression was highly reduced and restricted mainly to the internal zone of ME region in control rats (Fig. 4E and 5E). Using quantitative immunofluorescence staining intensity measurements, it was observed that GFAP-ir was significantly higher in both internal and external zone of ME region in IF-DR rats as compared to AL fed female (Fig. 4H and 4I) and male (Fig. 5H and 5I) rats.

The results of immunofluorescent staining were further confirmed by quantitative analysis of immunoblots and semiquantitative RT-PCR. GFAP and α-tubulin labelling in the representative samples in IF-DR and AL fed female and male rats from median eminence region of hypothalamus is shown in Fig. 4J and 5J, respectively. The mean value for the percentage of GFAP to α-tubulin ROD was increased in IF-DR female (Fig. 4K) and male (Fig. 5K) rats. The mRNA expression of GFAP was also observed to be upregulated in IF-DR female (Fig. 4L) and male (Fig. 5L) rats as compared to AL fed animals. The mean value of GFAP mRNA expression ratio to β-actin was significantly higher in IF-DR female (Fig. 4M) and male (Fig. 5M) rats.

### Expression of PSA-NCAM in AL and IF-DR Female and Male Rats

**Figure 6:**
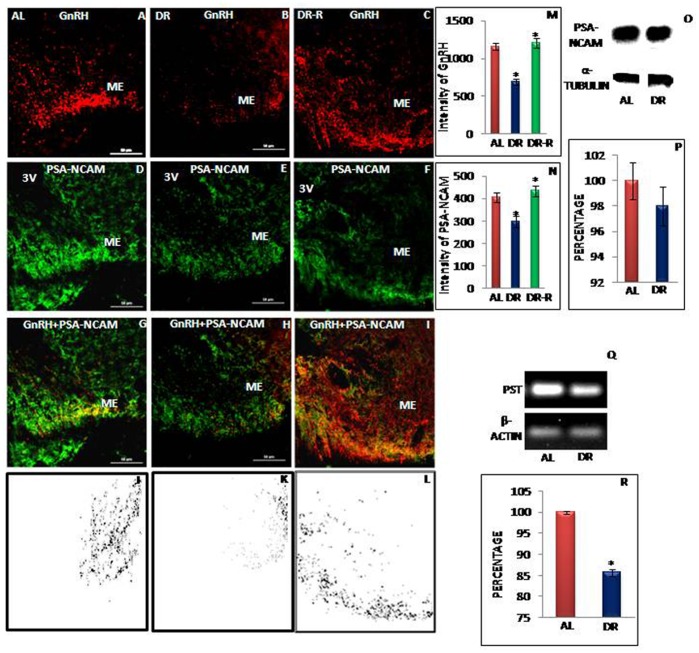
Representative immunofluorescent staining, Western blot and RT-PCR data of GnRH and PSA-NCAM in female rats. Confocal immunofluorescent images of 30 µ thick coronal sections from median eminence-arcuate (ME) region of ad libitum fed (AL), Intermittent dietary restricted (IF-DR) and DR-R female (n = 5 each) rat brain. The immunostaining in the ME region for GnRH and PSA-NCAM is shown for GnRH (A–C), PSA-NCAM (D–F) as well as dual immunofluorescence of GnRH and PSA-NCAM (G–I) for AL fed, IF-DR and DR-R animals, respectively. Both GnRH (A–C) and PSA-NCAM (D–F) immunostaining was reduced in IF-DR animals as compared to their corresponding control as well as DR-R female rats. (O) Western blot hybridization for PSA-NCAM and α-tubulin from median eminence region of AL and IF-DR female rats. (Q) RT-PCR results for PST and actin from median eminence-arcuate region of AL and IF-DR female rats. (M) depicts relative intensity measurement ±SEM of GnRH immunofluorescence, performed by an observer blind to the experiments. (N) depict relative intensity measurement ±SEM of PSA-NCAM immunofluorescence from ME region. (P, R) depict relative optical density ±SEM of PSA-NCAM western blot analysis and PST mRNA expression level from median eminence-arcuate region. *pValue<0.05. Scale bar = 60 µ (A–I).

**Figure 7:**
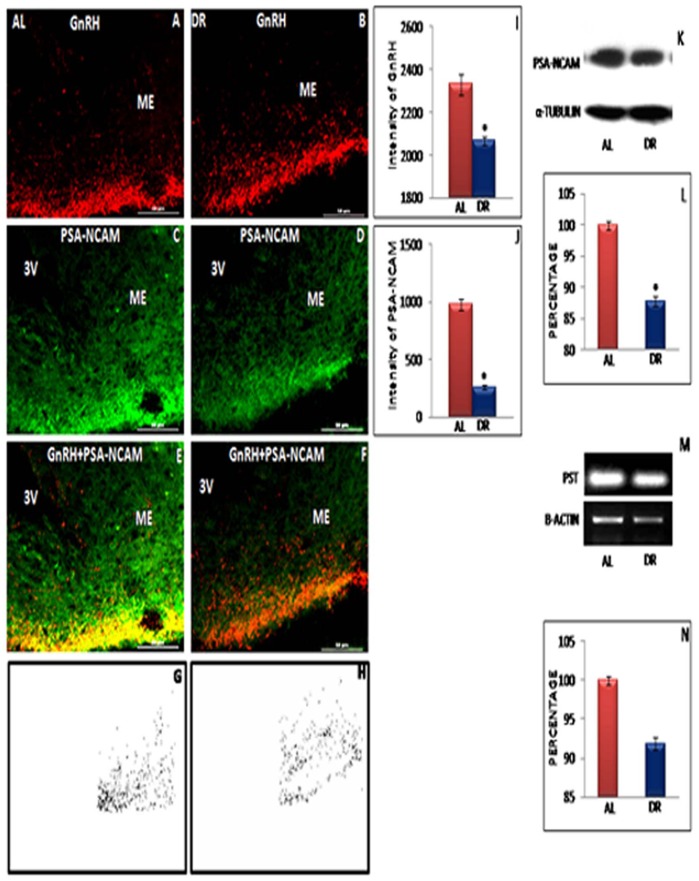
Representative immunofluorescent staining, Western blot and RT-PCR data of GnRH and PSA-NCAM in male rats. Confocal immunofluorescent images of 30 µ thick coronal sections from median eminence-arcuate (ME) region of ad libitum fed (AL) and Intermittent fasting-dietary restricted (IF-DR) male (n = 5 each) rat brain. The immunostaining in the ME region for GnRH and PSA-NCAM is shown for GnRH (A and B), PSA-NCAM (C and D) as well as dual immunofluorescence of GnRH and PSA-NCAM (E and F) for AL and IF-DR animals, respectively. Both GnRH (A and B) and PSA-NCAM (C and D) immunostaining was reduced in IF-DR animals as compared to their corresponding control rats. (K) Western blot hybridization for PSA-NCAM and α-tubulin from median eminence region of AL and IF-DR male rats. (M) RT-PCR results for PST and actin from median eminence region of AL and IF-DR male rats. (I) depicts relative intensity measurement ±SEM of GnRH immunofluorescence, performed by an observer blind to the experiments. (J) depict relative intensity measurement ±SEM of PSA-NCAM immunofluorescence from ME region. (L and N) depict relative optical density ±SEM of PSA-MCAM western blot analysis and PST mRNA expression from median eminence region. *pValue<0.05. Scale bar = 60 µ (A–F).

Earlier we have reported that PSA-NCAM-ir is associated primarily with the periphery of the GnRH axon terminals and regulated by excitatory and inhibitory inputs [26]. GnRH-ir in control and IF-DR animals is shown in Fig. 6 and 7. In control AL fed animals, PSA-NCAM-ir was intense and co-localized with a region of intense GnRH immunoreactive axon terminals near the perivascular space as evident from yellow color staining appearing in co-localized area (Fig. 6G and 7E). On the other hand, GnRH and PSA-NCAM co-expression was very much reduced in IF-DR female (Fig. 6H) and male rats (Fig. 7F). The immunostaining intensity of GnRH and PSA-NCAM from DR-R female animals (Fig. 6C, F, I) was similar to AL fed animals. Results of co-localization analysis shown in Fig. 6 and 7 further illustrate that GnRH axon terminals highly co-localize with PSA-NCAM in AL proestrous female rats (Fig. 6J) and AL male rats (Fig. 7G) as compared to IF-DR female (Fig. 6K) and male (Fig. 7H) rats, respectively. Quantitative immunofluorescence staining intensity measurement, further confirmed decreased expression of PSA-NCAM in IF-DR rats as compared to control group (Fig. 6N and 7J).

Immunofluorescent staining data was further supported by quantitative analysis by immunobloting and semiquantitative RT-PCR results. PSA-NCAM and α-tubulin labelling in the representative samples from median eminence region of hypothalamus in IF-DR and AL fed female (Fig. 6O) and male rats (Fig. 7K) and the mean value for the percentage of PSA-NCAM to α-tubulin ROD is shown in Fig. 6P and 7L. The mRNA expression of PST was significantly reduced in IF-DR female (Fig. 6Q) and male (Fig. 7M) rats as compared to AL fed animals. The mean value for the percentage of PST to β-actin is shown in Fig. 6R and 7N.

## Discussion

Feeding regimen of intermittent fasting-dietary restriction of three months duration reduced body weight and blood glucose level in both young (3–4 months) male and female Wistar strain rats and resulted in 19% and 34% loss in body weight in female and male rats, respectively. On the other hand, IF-DR regimen adversely affected the reproductive function as evident from disrupted estrous cycle and reduced ovarian weight observed in IF-DR female rats as compared to AL fed control animals, which showed normal estrous cyclicity. Estrous cycle was disturbed after about two weeks of initiation of alternate day feeding in female rats. The data further revealed that IF-DR regimen markedly enhanced serum estradiol in female rats and reduced testosterone level in male rats, whereas, serum LH concentration was found to drastically decrease in animals of both the sexes on IF-DR regimen. The current data suggests that IF-DR regimen adversely affects reproduction in young adult rats by disrupting estrous cycle in female rats as well as altering serum concentration of estradiol, testosterone and LH in both male and female animals. These results are supported by recent studies reporting significant reduction in body weight of ewes on food restriction and irregular and completely disrupted estrous cycle which were observed in 20% to 40% caloric restricted rats [32]–[34]. Similar studies in bull reported that low nutrition levels during calfhood adversely affected LH secretion and impaired testicular steroidogenesis as well as inhibited GnRH pulse generator and delayed puberty [7], [8]. Decrease in body weight and body mass index has also been reported in the anorexia nervosa patients as compared to control subjects [35]. Moreover meal related insulin secretion is altered in human subjects with anorexia [36].

Further, we observed sex-based difference in animals responding to IF-DR regimen as the reproductive alterations were more pronounced in IF-DR females as compared to their male counterparts. The heterogeneous hormonal response has also been observed in the patients with restrictive anorexia nervosa [37]. Several abnormalities have been reported in anorexia patients such as low T3 syndrome; decreased levels of free T4, IGF-I [38], and free testosterone [39]. Estrogen receptor-α (ERα) expressing inter-neurons have been shown to mediate estrogen feedback to GnRH neurons [40]. Estrogen exerts negative feedback effect by reduced serum LH through estrogen response element independent of estrogen receptor-α (ERα) signaling pathway [41]. Exogenous treatment of estradiol in sows and gilts is known to decrease hypothalamic GnRH, pulse amplitude and frequency of LH release [42]. These findings support the current data suggesting that increase in estradiol level in IF-DR female rats may lead to down regulation of GnRH and LH level via negative feedback. The reduced gonadotropin secretion may be due to the inhibitory effect of estrogen at the pituitary level and androgens at the hypothalamic level. Estrogens in the ingested food and anabolic steroids used by some athletes and body builders also show similar effect [43]. Significant decrease in mean plasma LH and testosterone pulse frequency has been reported in men after 48 h fasting period [44].

The energy status of an animal, depending on its dietary regimen/intake is known to regulate diverse physiological functions as well as alter estradiol and testosterone levels in males and females [45]. The relationship between dietary intake and reproductive capacity seems to be especially vulnerable to energy status in the female as is evident from disruption of estrous cyclicity within few days of initiation of the IF-DR regimen in the current investigation. A recent study suggests that reproduction involves higher energy expenditure in female animals as compared to male animals [46]. Moreover, the physiological functions of females such as pregnancy and lactation also demand considerable energy to nurture the embryos and newborns [47]. Several recent studies also support the probability of altered reproductive function and reduced LH pulse frequency during negative energy balance [1], [7]. The decreased serum testosterone level in IF-DR male rats may be responsible for reduced GnRH synthesis/secretion. The current data further strengthens the physiological basis of proposed link between nutritional/energy status and reproduction and their relationship that involves multiple regulatory molecules signaling at different levels of the reproductive axis.

The present study further elucidates alteration in the levels of metabolic fuel detectors/regulators such as leptin and hypothalamic ME-ARC region expression of NPY and kisspeptin. We observed significant reduction in serum leptin level in IF-DR rats of both the sexes. The decrease observed in the blood glucose level in rats on IF-DR regimen (with females showing more pronounced percentage reduction) may link these fuel detector’s function to inhibit the expression of GnRH and serum LH concentration. Welt et al [48] reported that treatment of leptin in leptin deficient subjects and undernutrition women with hypothalamic amenorrhea induced increased gonadotropins, LH and estradiol level, ovarian volume and the number of dominant follicles. Recent findings suggest that hypoleptinaemia is the critical signal responsible for inhibition of ARC Kiss-1 and LH during negative energy state [49]. Moreover, plasma concentration of leptin and insulin were reduced in food restricted OVX ewes for 4 months [50]. In the current study, animals of both the sexes on IF-DR regimen showed increase in NPY-ir and its mRNA expression, whereas, serum leptin level and hypothalamic kisspeptin expression were down regulated. The leptin receptor mRNA is co-expressed with kisspeptin and NPY in the arcuate region of hypothalamus and the administration of leptin is known to reduce NPY and upregulates kisspeptin expression [3]. NPY up regulation observed during IF-DR regimen may be due to reduced serum leptin level as it has been reported to act as a metabolic signal to regulate NPY expression [36]. Previous studies have also revealed that leptin treatment upregulates kisspeptin mRNA expression in mouse hypothalamic cell line [17]. The presence of leptin receptor on kisspeptin neurons of arcuate region also demonstrate that these cells respond to peripheral leptin level [11]. These findings support our observations that the reduced circulating leptin level in IF-DR animals may be responsible for down regulating kisspeptin and enhancing NPY expression in arcuate nucleus and thus link the energy status through these metabolic sensors in hypothalamus.

NPY fibres make synapse with the dendrites and cell bodies of GnRH neurons in preoptic area and GnRH neuron terminals in ME region of hypothalamus [51], thus suggesting that NPY may be acting as a link between energy status and GnRH pulse generator. A recent finding also reported that caloric restriction up regulated NPY expression in hypothalamus [35]. The current and previous findings suggest that increased NPY expression in animals on dietary/caloric restriction may inhibit the release of GnRH from median eminence and thus affecting the HPG axis [52], [53]. These observations may also explain the basis of infertility resulting from restricted food intake by women under conditions such as anorexia nervosa and exercise-induced amenorrhea. We further observed down regulation in kisspeptin-ir and its mRNA expression in IF-DR male and female rats. Kisspeptin is reported earlier to act on HPG axis by stimulating GnRH neurons thus inducing its secretion [54] and reported reduction in KISS-1 mRNA expression after fasting [17], [55]. Kisspeptin has distinct action on GnRH release, mediating the negative feedback of sex steroids on GnRH secretion via neurons in the arcuate region of hypothalamus and positive feedback of sex steroids via anteroventral paraventricular thalamic (AVPV) neurons [19] and promotes GnRH secretion via G-Protein coupled receptor (GPR-54) [56]. Mutation in GPR-54 receptor causes hypogonadotrophic hypogonadism in rodents and humans [57]. Both fasting and lactation have been reported to reduce KISS-1 and GPR54 mRNA expression [17].

The present study further investigated the effect of IF-DR regimen on the morphological remodeling of GnRH neuron terminals in the ME region of hypothalamus as our lab has earlier reported that GnRH axon terminals near perivascular space are known to undergo remodeling in order to facilitate release of neurohormone in hypophysial portal circulation [20], [58]. Animals on IF-DR paradigm showed decrease in expression of GnRH and PSA-NCAM in ME region of hypothalamus in both male and female rats which recovered to near control levels in DR-R female animals. Further the astro-glial cells showing expression of GFAP were also seen to extend their processes between GnRH axon terminals in the outer zone of ME region in rats on IF-DR regimen. These findings are supported by recent reports from our lab and confirm the functional role of PSA-NCAM in dynamic remodeling of astro-glial processes and GnRH axon terminals near perivascular space to facilitate the release of GnRH in the hypophysial portal system during proestrous phase of reproductive cycle [20], [26], [59].

In conclusion, the data shown in table 2 may help to understand how the individual factors such as metabolic fuel, sex hormones, leptin, NPY and kisspeptin act as neuroendocrine regulators to link reproductive dysfunction to the perturbations in energy balance. Changes in the expression level of these molecules adversely affected GnRH expression and its release from the ME region of hypothalamus in both the male and female rats on IF-DR regimen, thus supporting the proposed hypothesis of a central link between reproduction and energy status [3], [60]. It may be suggested that the reduced leptin levels in IF-DR animals conveys the nutritional signal to arcuate nucleus of hypothalamus through the metabolic sensors such as NPY and kisspeptin, and further the alterations observed in their expression may be important factors in disrupting the function of HPG axis in response to negative energy balance. Moreover, the current results suggests that the GnRH neurons which occupy a master position in HPG axis, become target of many central and peripheral origin metabolic regulators directly or indirectly thus compromising reproductive functions in the face of energy status. The current data may help to understand the clinical basis of nutritional infertility observed in patients of anorexia nervosa and hypothalamic amenorrhea due to metabolic stress, excessive exercise, undernutrition etc.

**Table 2:** Summarized results showing parameters studied and the comparison between AL fed and IF-DR animals.

Parameters studied	IF-DR as compared to AL group
Body Weight	−
Blood Glucose	−
Ovarian Weight	−
Estradiol (Female)	+
Testosterone (Male)	−
Leutinizing Hormone	−
Leptin	−
NPY	+
Kisspeptin	−
GnRH	−
GFAP	+
PSA-NCAM	−
